# Degradation kinetics of cold plasma-treated antibiotics and their antimicrobial activity

**DOI:** 10.1038/s41598-019-40352-9

**Published:** 2019-03-08

**Authors:** Chaitanya Sarangapani, Dana Ziuzina, Patrice Behan, Daniela Boehm, Brendan F. Gilmore, P. J. Cullen, Paula Bourke

**Affiliations:** 1 School of Food Science and Environmental Health, Technological University Dublin, Dublin, Ireland; 2 School of Chemical and Pharmaceutical Sciences, Technological University Dublin, Dublin, Ireland; 3 School of Pharmacy, Queens University Belfast, Belfast, BT9 7BL United Kingdom; 4 School of Chemical and Biomolecular Engineering, The University of Sydney, Sydney, Australia

## Abstract

Antibiotics, such as ofloxacin (OFX) and ciprofloxacin (CFX), are often detected in considerable concentrations in both wastewater effluents and surface water. This poses a risk to non-target organisms and to human health. The aim of this work was to study atmospheric cold plasma (ACP) degradation of antibiotics in water and meat effluent and to explore any residual antimicrobial activity of samples submitted to the plasma process. The results revealed that ACP successfully degraded the studied antibiotics and that the reaction mechanism is principally related to attack by hydroxyl radicals and ozone. According to the disk diffusion assay, the activity of both antibiotics was considerably reduced by the plasma treatment. However, a microdilution method demonstrated that CFX exhibited higher antimicrobial activity after ACP treatment than the corresponding control revealing a potentially new platform for future research to improve the efficiency of conventional antibiotic treatments. Importantly, short-term exposures to sub-lethal concentrations of the antibiotic equally reduced bacterial susceptibility to both ACP treated and untreated CFX. As a remediation process, ACP removal of antibiotics in complex wastewater effluents is possible. However, it is recommended that plasma encompass degradant structure activity relationships to ensure that biological activity is eliminated against non-target organisms and that life cycle safety of antibiotic compounds is achieved.

## Introduction

In recent years, there has been widespread and increased usage of antibiotics. Antibiotics are administered to humans and animals for control of bacterial infections, but are also widely used in cancer treatment and as growth promotion agents in some regions. Non-target environmental organisms are affected, resulting in risks of ecosystem disruption^1^. In light of the need for careful antimicrobial stewardship due to the pervasive threat of antimicrobial resistant microorganisms, the environmental release of effluents containing antibiotics or their residues is of concern. Due to their poor degradation in sewage treatment plants (STPs), these antibiotics often find their way into water sources^2^. It has been observed that wastewater treatment plants (WWTPs) are not equipped with the specific facilities for the removal of many pharmaceuticals, especially antibiotics^3^. Fluroquinolones (FQs), such as ofloxacin (OFX) and ciprofloxacin (CFX), are broad-spectrum antibiotics, which are widely used for respiratory and bacterial infections^4^. Among other FQs, OFX and CFX are commonly detected in WWTPs effluents^5^, surface waters^6,7^ and sea waters^8^. The ranges of concentrations for CFX in WWTP effluents have been reported to be as high as 5.6 mg/L^9^ and in hospital effluents in a range of 3–87 mg/L^10^. Moreover, the low bio-degradability of quinolones makes conventional biological treatment ineffective for their removal. In conventional wastewater treatment, prolonged exposure to antibiotics could lead to the emergence and evolution of antibacterial resistance^11^. Additionally, several reports conclude that these antibiotics and their residues pose a substantial toxic risk to aquatic organisms^12^. Current research efforts aim to develop new and efficient approaches to transform these chemical agents to pharmaceutically less active and more biodegradable species. Several authors have reported on the application of advanced oxidation technologies (AOP’s)^13^ including ozonation^14^, photocatalysis^15^, which have been proposed to remove antibiotic compounds^11,16–18^. Although these techniques, such as ozonation and combinations of AOPs lead to the degradation of many organic compounds in aqueous solutions, this does not necessarily mean that their total mineralization will be achieved^19,20^. Dantas *et al*.^21^ and Rosal *et al*.^22^ showed that ozonation may generate oxidation intermediates and enhance the toxicity for aquatic life. Contact with ozonated water resulted in inhibition of the reproduction of the annelid *Lumbriculus variegatus* due to the formation of toxic oxidation by-products^23^. Recently, atmospheric cold plasma (ACP) has emerged as a novel AOP technology because of its efficient degradation efficiencies and environmental compatibility^24^. ACP is composed of several excited atomic, molecular, ionic, and radical species, coexisting with numerous reactive species, including electrons, positive and negative ions, free radicals, gas atoms, molecules in the ground or excited state, UV light, shockwave and pyrolysis, which contribute to chemical and physical effects that can have an impact on organic pollutants decomposing them into more environmentally friendly compounds^24–26^. ACP has been efficient in the removal of dyes^27^, pesticides^28,29^, aflatoxins^30^ and pathogenic bacteria^31,32^. Currently, plasma technology is widely researched for the generation of functionalized solutions for biological decontamination of liquids. In plasma treated solutions reactive oxygen species (ROS), such as the hydroxyl (•OH·) radical, atomic oxygen (O), ozone (O_3_), and hydrogen peroxide (H_2_O_2_), play a major role in the inactivation processes^33^, although enhanced antimicrobial potential associated with an increase in nitric oxide (NO) radical concentration was also reported thereby providing new insight into the role of reactive nitrogen species (RNS)^34^. However, limited studies have been reported on the potential of plasma for the removal of antibiotics^35,36^. Lou *et al*.^37^ successfully achieved the degradation of antibiotics from contaminated soil. The effectiveness of a dielectric barrier discharge (DBD) plasma to degrade antibiotics in water systems was investigated by Kim *et al*.^38^ and Magureanu *et al*.^39^. Few reports are published on the identification of the degradation products and elucidation of the mechanisms governing their generation and their toxicity. It is important to know whether an intermediate product retains the biological effects of the parent compound or whether new and undesired biological effects are developed^12^. On the other hand, most of these studies on antibiotic degradation have been carried out in the absence of effluent organic matter that is generally present in wastewaters. Use of simulated or model effluents and identification of intermediate products and their toxicity studies could advance the suitability of cold plasma technology for wastewater treatment.

The objective of this work was to study the efficacy of atmospheric air plasma for the degradation of antibiotics in different liquid matrices, such as water and a model meat effluent. We aimed to elucidate the intermediate or transformed products in order to propose a reaction pathway. Furthermore, the antimicrobial property of the treated antibiotic solutions was also assessed with the potential for adaptive responses to any breakdown products in addition to exploring the possibility for enhanced biological safety.

## Results and Discussion

### Evaluation of degradation efficiency and kinetics

Ofloxacin (OFX) and ciprofloxacin (CFX) are among the most frequently detected antibiotics in the environment^5–8^. Although of the same chemical class, these antibiotics differ slightly in their molecular structure, which in the current research allowed reporting on the intrinsic factors influencing the efficacy of ACP for the removal of antibiotics from the environment.

**Figure 1:**
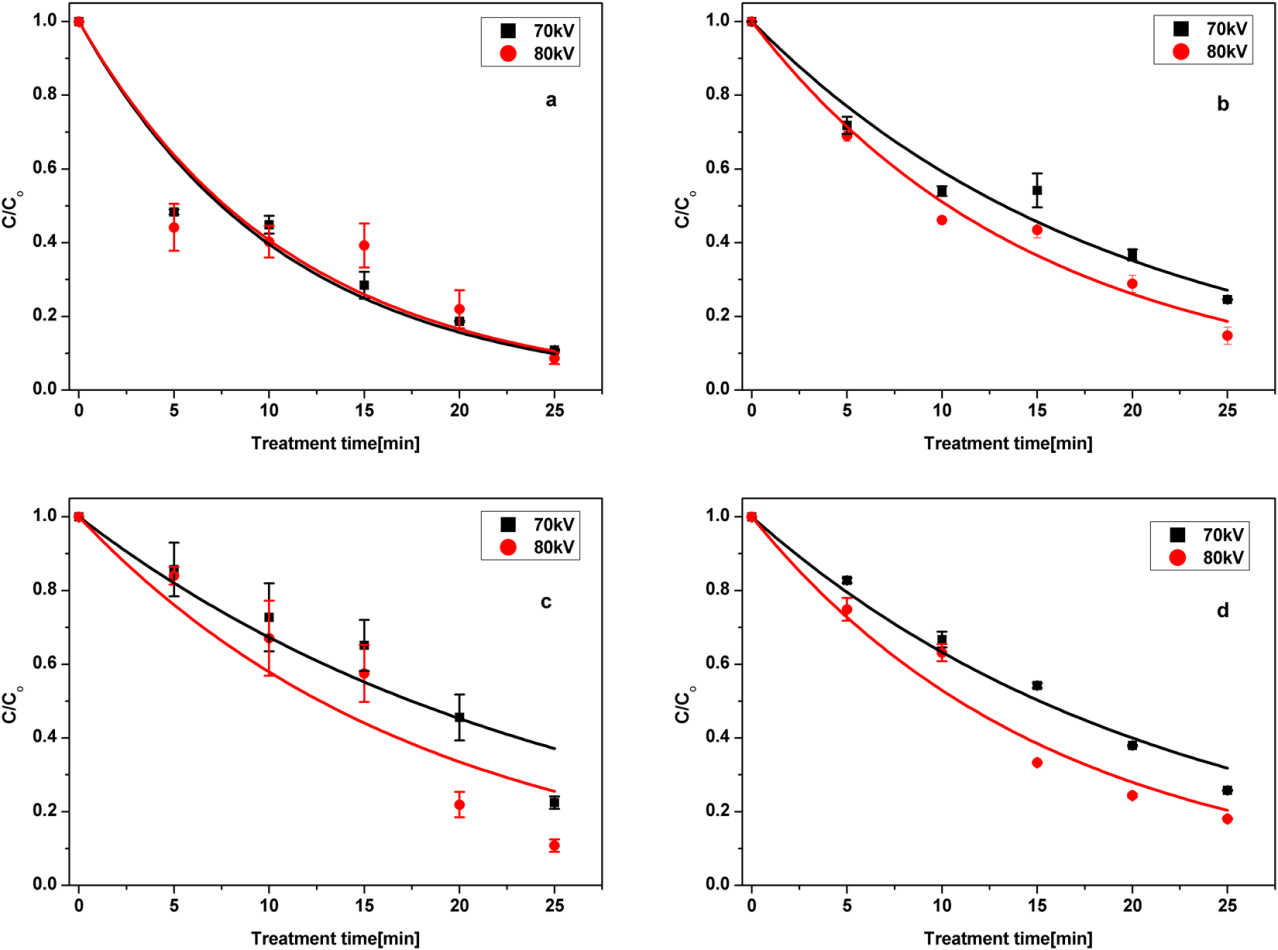
Degradation kinetics of antibiotic removal (**a)** OFX in water (**b**) OFX in meat effluent (**c**) CFX in water and (**d**) CFX in meat effluent at initial concentration of 10 mg l^−1^.

Atmospheric air plasmas can generate many chemical reactions, which are responsible for generating various active chemical species at ambient conditions, such as negative and positive ions, neutral molecules (H_2_, O_2_, O_3_, and H_2_O_2_), and free radicals (•OH, •O, •H, and HO_2_^•^). Among these species, ozone (O_3_), hydrogen peroxides (H_2_O_2_), hydroxyl radicals (•OH), as well as RNS (peroxynitrites, nitrogen oxides) are generally identified as the main active species responsible for the degradation of antibiotics^40–42^, although the role of other reactive species may also be important in the degradation process through other chain reactions. In the current work, the concentration profiles revealed that the degradation depends on the applied voltage (Fig. 1). Increasing the voltage from 70 to 80 kV increased the plasma degradation efficacy of antibiotics in water from 75% to 89% for CFX and from 88 to 92% for OFX, respectively. This can be attributed to generation of more reactive species. It was also observed that the degradation of antibiotics was dependent on the treatment time with higher degradation rates achieved after longer plasma exposure. The degradation kinetics of antibiotics can be explained by a first-order kinetic model (model parameters can be found in Supplementary Table S1). The rates of reaction were increased from *k* = 0.039 to *k* = 0.054 min^−1^ with an increase in applied voltage from 70 kV to 80 kV.

The degradation efficacies and the reactive species generated are influenced by many factors, such as input energy, the nature of the gaseous atmosphere as well as pH, conductivity, temperature of the solution, chemical structure of the contaminant and the solution matrix^43^. In the current work, the influence of the solution matrix was studied to reflect more realistic challenges. The degradation efficacy of ACP was influenced by the presence of meat organic matter, which decreased the efficacy by 10% for all applied voltages as compared to the water samples. This is due to the competition between antibiotics and the organics present in the model meat effluent, where the plasma generated active species are consumed not only in reactions with antibiotics, but also in reactions with the organic matter and antibiotic degradants. During plasma treatment, the active species, such as ozone, hydrogen peroxide and hydroxyl radical, react with unsaturated bonds and aromatic rings of the organic compounds (proteins, carbohydrates, fat) leading to the splitting of bonds and the dissociation of the rings, following the Criegee mechanism^44^. The removal efficiency may be characterized by the amount of contaminant degraded per unit of energy (yield). The yield can be influenced by many factors, including system design, the type and the concentration of the compound. For the antibiotic solutions studied, a decrease in energy yield values was observed with increase in time and voltage. With increase in voltage, the energy yield has reduced from 328 × 10^−6^ to 221 × 10^−6^ g/kW h and from 204 × 10^−6^ to 103 × 10^−6^ g/kW h for CFX and OFX in water, respectively.

**Figure 2:**
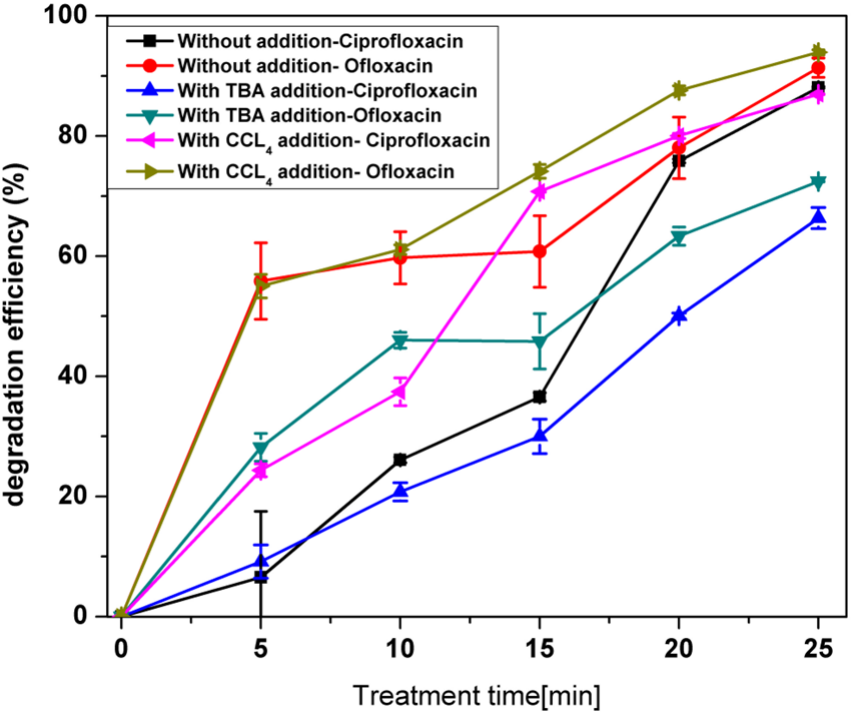
Effect of radical scavenger’s tertiary butanol alcohol (TBA) and Carbon tetra chloride (CCL_4_) on degradation of efficiencies of antibiotics in water at initial concentrations of 10 mg l^−1^.

ACP generates several oxidizing species: radicals (•OH, .H) and molecules (O_3_, H_2_O_2_, etc.) of which the hydroxyl radical is considered a very strong non-selective oxidant (Eo = 2.85 V/SHE). To identify the role of these radicals, experiments were conducted by adding radical scavengers. As shown in Fig. 2 the degradation efficiency of CFX and OFX in water was 88% and 91%, respectively. In the presence of the radical scavenger tertiary butyl alcohol (4 mmol l^−1^), an efficient hydroxyl radical quencher, the degradation efficiency dropped to 66% for CFX and 72% for OFX. The reaction of the hydroxy radical with most organic pollutants occurs either by hydrogen abstraction with saturated aliphatic hydrocarbons and alcohols, or electrophilic addition with unsaturated hydrocarbons^45^. Interestingly, higher removal efficiencies were achieved in the presence of CCl_4_, which could quench •H in antibiotic solution. The removal efficiency increased to 90.5% for CFX and 93% for OFX by adding 0.5 ml CCl_4_ to the antibiotic solutions. This may be attributed to a decrease in •H concentration, which inhibits the frequency of recombination between the hydrogen atom with the hydroxyl radical^46^.

**Figure 3:**
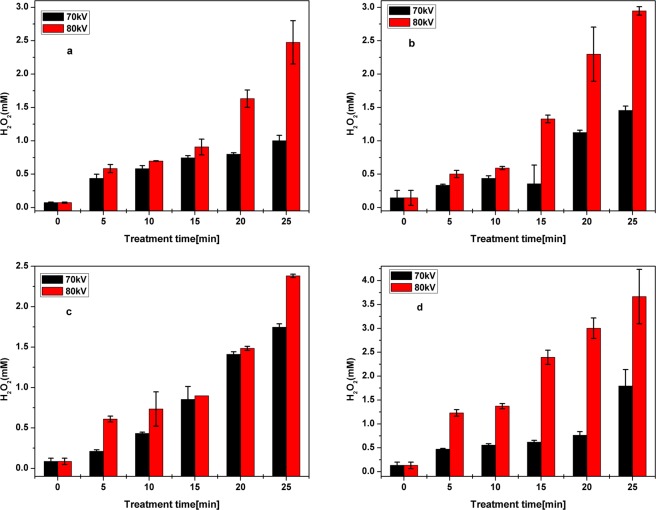
Variation of H_2_O_2_ concentration in plasma treated antibiotic solutions (**a**) OFX in water (**b**) OFX in meat effluent (**c**) CFX in water and (**d**) CFX in meat effluent.

Hydrogen peroxide (Eo = 1.77 V/SHE) is one of the most reactive species formed in gas-liquid plasma system. It is generally formed by the recombination of generated hydroxyl radicals in oxygen free water^45,47^. Hydrogen peroxide under ambient conditions is relatively stable in aqueous phosphate buffer solution with the reported stability of several weeks^48^. This can also be used as an efficient indicator of the •OH generation in the DBD contained plasma reactor. In order to evaluate the efficacy of the DBD reactor, the formation of H_2_O_2_ in antibiotic solutions was quantified (Fig. 3). It can be observed that the concentration of H_2_O_2_ in the liquid phase generated by the DBD reactor is dependent on the treatment time and applied voltage. The concentrations of H_2_O_2_ ranged from 0.174 to 2.7 mM for antibiotics solutions in water with slightly higher values (0.21 to 3.6 mM) observed for antibiotics in the meat effluent. Similar results have been reported by previous authors^49,50^. Focusing on the degradation of antibiotics by DBD plasma, Hama Aziz *et al*.^43^ reported that the presence of pharmaceuticals or organic matter can enhance the formation of H_2_O_2_.

The presence of oxygen in the gas atmosphere induces the production of reactive species like oxygen radicals and ozone. Ozone is one the most stable active species generated in DBDs with a high oxidation potential of 2.02V^51^. The interaction between ozone and organic compounds can be either direct or indirect where decomposition occurs through a series of reactions. It was believed that ozone formed in the gas phase during the discharge is dissolved in deionized water until saturation. In this study, the gas phase ozone concentrations measured after 10 min of plasma treatment were found to be 2100 and 3200 ppm (within ± 10% errors) for applied voltages of 70 and 80 kV, respectively. In our study, very low concentrations of dissolved ozone were achieved (data not shown). This is probably due to the rapid consumption of generated ozone during the degradation process of the antibiotics. Several authors have explained the formation of •OH radicals during the discharge in a chain reaction with ozone in water^52^. The lower dissolved ozone values can also be attributed to the consumption of ozone by the antibiotic itself and their intermediate products.

**Figure 4:**
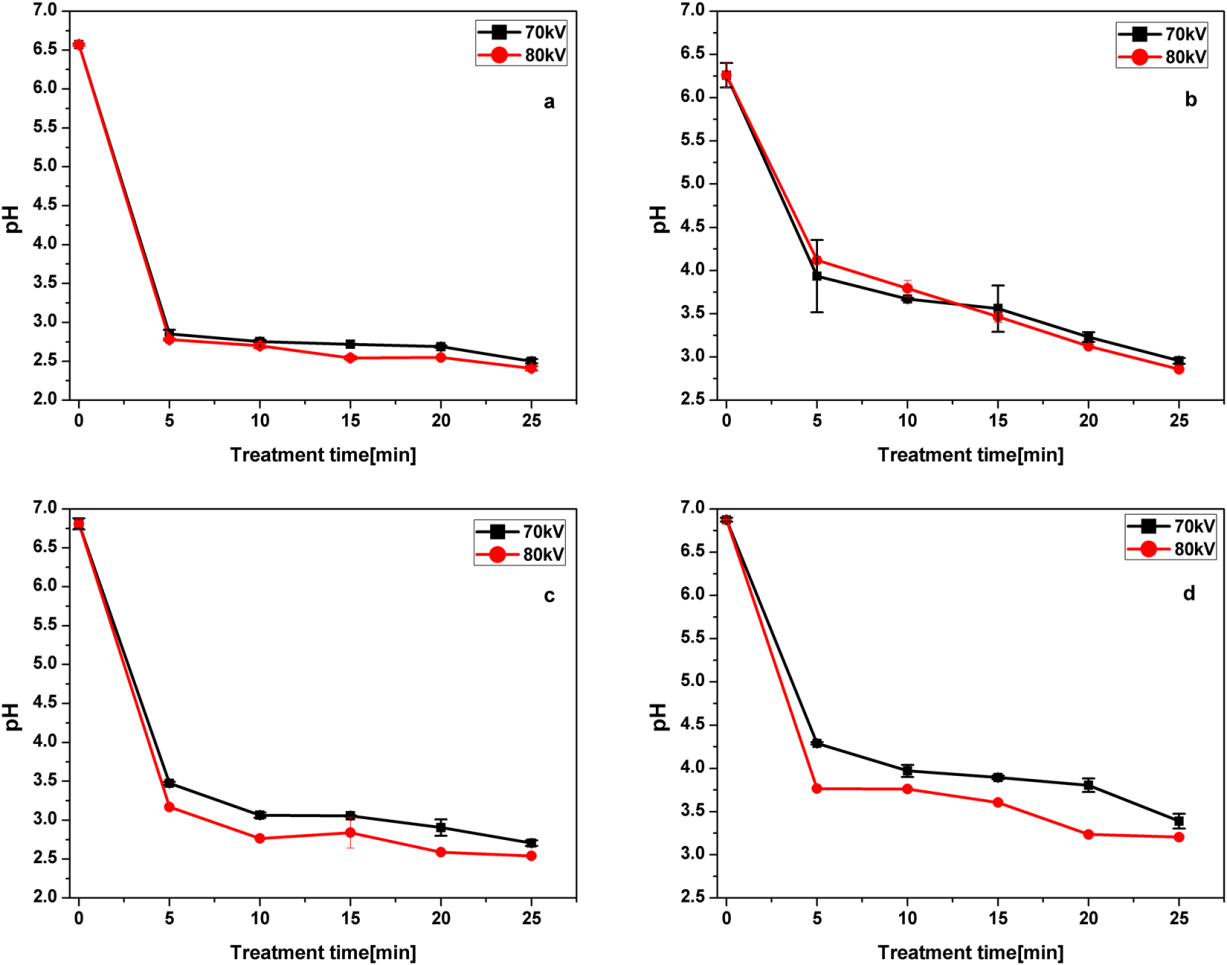
Evolution of pH in plasma treated antibiotic solutions (**a**) OFX in water (**b**) OFX in meat effluent (**c**) CFX in water and (**d**) CFX in meat effluent.

**Figure 5:**
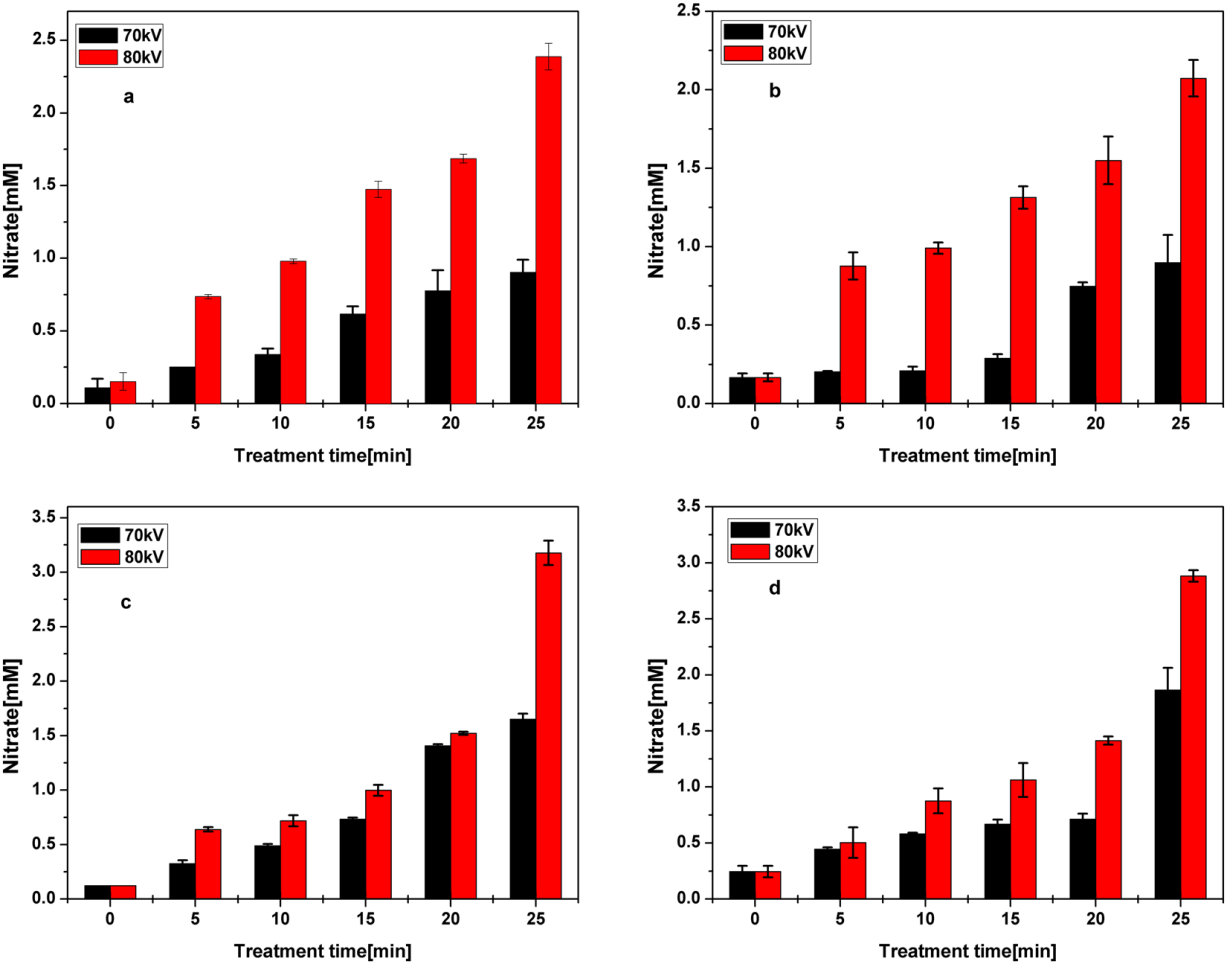
Variation of nitrate concentrations in plasma treated antibiotic solutions (**a**) OFX in water (**b**) OFX in meat effluent (**c**) CFX in water and (**d**) CFX in meat effluent.

The variation of pH values for the solution after plasma treatment process is shown in Fig. 4. The pH value was lowered with increasing treatment time for all antibiotic solutions. After the first 5 min of treatment the pH values dropped to less than 4.5 and subsequent increases in treatment time resulted in a slow decline. This variation of pH values in the antibiotic solutions is caused by the formation of several specific acidic substances, such as nitric acid and nitrous acid during plasma treatment in air^27^. Also, a certain contribution to the pH variation during the plasma treatment may come from carboxylic intermediates, produced from the degradation of antibiotics. To confirm the acidification of the antibiotic solutions caused by air plasma the treated solutions were analyzed for the presence of nitrates, nitrites and several carboxylic acids. The concentrations of nitrate in the antibiotic-water solutions after 25 min of plasma treatment were found to be 0.98 mM and 2.38 mM for applied voltages of 70 and 80 kV, respectively (Fig. 5). An increase in nitrate values was observed with increases in treatment time and applied voltage. Similarly, the formation of high nitrate concentrations in the solution were reported for gliding arc plasma treatments^53,54^. Such increases in nitrates cause a drop in pH. Moreover, no nitrites were detected in the present study, which is due to the easy conversion of nitrite to HNO_2_ under acidic conditions. Nitrates formed under acidic conditions undergo several reactions to form peroxynitrite acid^55^. Oxidative ability of reactive nitrogen species is lower than reactive oxygen species, as they are formed by consuming the stronger oxidant •OH, which in turn converts them to HNO_3_^56^.

**Figure 6:**
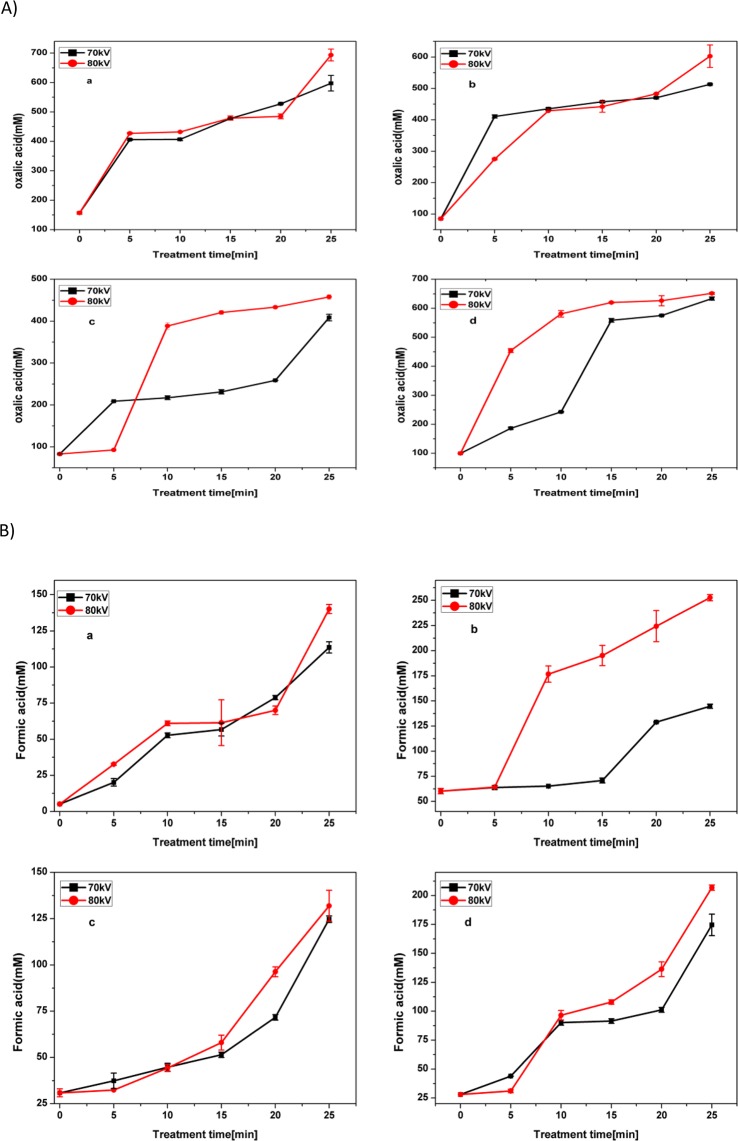
(**A) -** Variations of concentrations of oxalic acid in plasma treated antibiotic solutions (a) OFX in water (b) OFX in meat effluent (c) CFX in water and (d) CFX in meat effluent. (**B) -** Variations of concentrations of formic acid in plasma treated antibiotic solutions (a) OFX in water (b) OFX in meat effluent (c) CFX in water and (d) CFX in meat effluent.

Several authors have reported that the ultimate oxidation by-products of pharmaceuticals are low-molecular weight carboxylic acids, such as oxalic acid, acetic acid, formic acid^57,58^. However, the toxicity of the degradation by-products may still be a matter of concern. It is known that ACP treatment of aqueous organic pollutant results in the formation of several intermediate products, such as carboxylic acids, formic acid and oxalic acid. The concentrations of formic and oxalic acids were determined using ion chromatograph and are presented in Fig. 6(A,B), respectively. It can be observed that the concentration of both organic acids increased with treatment time and all applied voltages. Similar results in the formation of organic acids as by-products have been reported by Vasquez *et al*.^59^ in plasma-treated aqueous antibiotic solutions. The formation of carboxylic acids resulted in slow mineralization and low total organic carbon (TOC) removal values after plasma treatment.

### Degradation mechanism of antibiotics

The plasma degradation of organic contaminants takes place through several reactions. In the presence of air/oxygen the mechanism is not only based on the hydroxyl radical or ozone formation but also based on photo-oxygenation, photo-isomerisation or photo hydrolysis^28,51^. The carbon-centered radicals may yield peroxy radicals, which can be further decomposed to form corresponding oxidation products. Other mechanisms include the formation of superoxide radicals, which could further recombine, rearrange or hydrolyze to final products. Like photolytic or photocatalytic processes, the organic contaminant during plasma treatment undergoes reactions through excitation or ionization^60^. These excited contaminants may also quench molecular oxygen with formation of singlet oxygen. It is reported that oxygen in its singlet oxygen state (E_0_ = 1.77 V) is more reactive compared to the molecular oxygen. Consequently, singlet oxygen is significantly more electrophilic^61^. This singlet oxygen can react rapidly with unsaturated carbon-carbon bonds neutral nucleophiles, such as sulfides^62^ and amines^60^, as well as with anions, and form an hydroperoxide as an intermediate compound.

In order to understand the antibiotic degradants present post plasma treatment, the changes of the LC–MS/MS chromatograms in full scan mode were examined. Samples taken at the time when the greatest range of by-products appeared were further analyzed by comparison to the literature to identify their molecular structures. The proposed pathways of these intermediate products are expected to include multiple routes due to the presence of several reactive sites in the parent compound but in the present study the occurrence of two oxidation mechanisms by both molecular ozone and hydroxyl radicals was considered. Based on the experimental data and literature survey the degradation of the quinolone group of antibiotics occurs as follows: the plasma generated active species, such as •OH and O_3_, attack the carboxyl group of the quinolone moiety at the first place, followed by subsequent attack of the piperazinyl substituent and oxazinyl substituent^63^. In our study, no transformation products were found corresponding to the degradation of the oxazinyl group. These pathways are in line with work published by Carbajo *et al*.^12^.

The proposed degradation mechanism of OFX can be found in Supplementary Fig. S2. The MS/MS spectrum of OFX with a product ion at m/z 362.1 showed three significant product ions at m/z 261.1034, 318.2 and 344.1. The product at m/z 344.1 is due to the loss of H_2_O and the loss of 1- methylpiperzine group forms fragment ion at m/z 261.1. The MS spectrum of plasma treated samples showed two products ions at m/z 378 and m/z 394. These ions might be formed due to electrophilic reaction between plasma active species, such as ozone, and OFX. This electrophilic substitution reaction at the C_12_ position of OFX leads to TP1 (m/z 378) and a subsequent similar reaction at the C8 position may yield TP2 (m/z 394). Similar intermediate products have been identified by Tay and Madehi^3^ in the ozonation of OFX in water. In this study, another product with m/z 380 has been identified as TP3. The oxidation of quinolone moiety through breaking of double bonds leads to the formation of TP3. The peak identified in the MS/MS at m/z 354 is an intermediate product TP4, likely formed by the selective reaction of hydroxyl radical with an electron rich site. Formation of TP4 commences with hydrogen abstraction at C3 by •OH. Subsequent hydrogen abstraction by •OH forms the intermediate product. Therefore the intermediate product formed undergoes intermolecular rearrangement through cleavage of C2-C3, which in turn undergoes a series of radical reactions and further hydroxylation to form TP4^3^. In OFX degradation, pathway 2 (Supplementary Fig. S3), the formation of TP5 (m/z 318) could be attributed to the attack of the carboxyl group of quinolone moiety by plasma generated •OH. This is due to the fact that the ionized OFX molecule possesses a negative charge at the carboxyl group, thus •OH is more prone to attack at carboxyl group than other species^59^. The attack by plasma species, particularly •OH, on the piperazinyl substituent can evolve two degradation pathways, i.e., α-abstraction and β-abstraction^63^. The β-abstraction of OFX undergoes several reactions by the addition of hydroxyl groups. These series of reactions with the elimination of methylene groups leads to the formation of TP6 (m/z 261). After the cleavage of the piperazinyl ring plasma active species likely attack the oxazinyl substituent, which finally leads to the formation of TP7 (m/z 221). The α-abstraction starts with the attack of the methylene group (demethylation) and the piperazine core. Demethylation of the piperazinyl ring leads to the formation of TP8. TP9 is attributed to ozone attack on the N4 atom. The main transformation product,TP10, is formed due to the addition of a hydroxyl radical at C7. It can also be considered as an oxidation product. The peak identified in the MS/MS spectra TP11 with m/z 376 is an intermediate product, likely to be a keto derivative, formed by plasma oxidative reactions^12^. TP12 (m/z 336) is proposed to be formed by the attack of the piperazine ring by plasma active species. The fragments at m/z 168(TPB) and m/z 157 (TPC) are also seen in the MS spectrum, as this could be formed through chain fragmentation of OFX. According to Shen *et al*.^64^, fluoroquinolones require the carboxyl group (-COOH) for antimicrobial activity. Therefore, it can be observed that the degradation of OFX leads to low molecular weight compounds such as m/z 157 with no antimicrobial activity.

The proposed degradation mechanism of CFX can be found in the Supplementary Fig. S4. The MS/MS spectrum of CFX with a product ion at m/z 362.1 showed four significant product ions at m/z 231.1, 245, 294, and 314. Similar to OFX, several intermediate products (IP) have been identified from the MS/MS spectra of CFX. On the other hand, various peaks of by-products appeared whose intensity increased with time and which again disappeared with further increase in plasma doses^35^. This is due to the fact that CFX was first degraded into various intermediates, and then mineralized if sufficient treatment time is provided. The two major peaks IP1 (m/z 330) has been identified in MS/MS spectra. This product might be formed by the substitution reaction of the fluorine atom by the hydroxyl radical. Similar structures has been proposed by Salma *et al*.^65^ in the photolytic and photo catalytic treatment of CFX. The formation of IP2 (m/z 346) can be proposed by the addition of a hydroxyl group at the piperazine ring of IP1^66^. The degradants IP3(m/z 360) and IP4(m/z 332) are formed due to the addition reactions of plasma treatment. It can be hypothesized that plasma species attack the piperazine ring of CFX or it could undergo photo-oxygenation yielding peroxy radicals (peroxy-piperazine rings). This intermediate product undergoes an initial attack of •OH via hydrogen abstraction leading to the opening of the piperazine ring, which is converted to the ketone derivative IP4 and further oxidation results in carboxylic derivative^65^. Difluorination of CFX and addition reactions similar to IP3 would result in the formation of IP5 (m/z 344), subsequent reaction would result in IPA (m/z 245). The intermediate products identified in pathway II (Supplementary Fig. S5) were found in most of the samples, which occur especially under neutral and acidic conditions with preservation of the fluorine atom^65,66^. The product IP6 (m/z 348) could be the hydroxylation product of CFX, formed via direct reaction between plasma active species, such as ozone and hydroxyl radicals. The formation of monohydroxy BPA was further confirmed by the fragmented peak ions at m/z 330 and m/z 245. This reaction further proceeded to yield IP7 (m/z 364). The formation of IP7 is attributed to the attack of •OH formed and the addition of an hydroxyl radical to the piperazine ring^67^. The spectra at m/z 346 is an intermediate product IP8, likely to be a keto derivative, formed by the selective reaction of ozone with an electron rich site without any cleavage of the piperazine ring^65,67^. This IP8 on further oxidation follows a ring opening mechanism forming dicarboxylic acids. A similar degradation mechanism has been observed in air plasma treatment of endocrine disruptors^49,57^. The product IP9 follows a similar reaction to IP3 but with the elimination of the fluorine atom. The cleavage of the piperazine ring followed by the loss of CO yields the product IP10. This oxidation follows a fragmentation pattern to yield IP11 (m/z 306) and IP12 ( m/z 263). Previously Torniainen *et al*.^68^ identified similar products to the final degradation product of CFX at low pH. According to the proposed reaction pathway, plasma treatment of antibiotics OFX and CFX may not undergo complete degradation. However, the primary structure was transformed into low molecular weight compounds upon higher plasma doses. Overall it can be observed that reactions at the piperazine group occur by molecular ozone where reactions at quinolone moiety are primarily a consequence of radical reactions. The changes in the piperazine group may not decrease the activity, but changes in the carbonyl and carboxyl groups at the quinolone moiety, which are essential for binding at the DNA gyrase thereby decreasing the overall activity of antibiotics.

### Antibacterial activity

**Figure 7:**
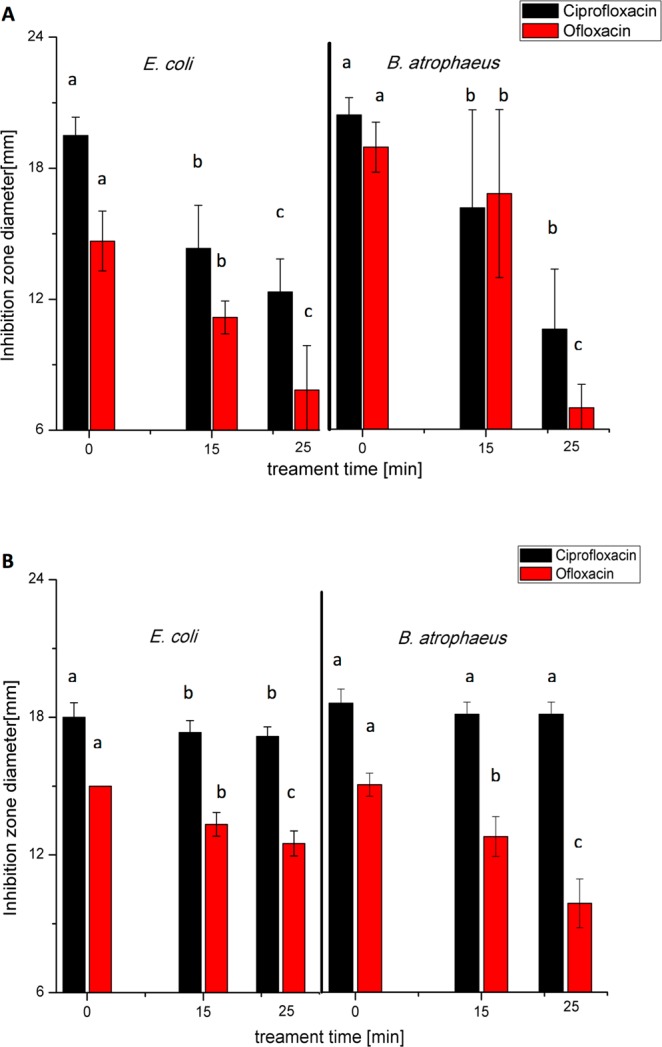
Antimicrobial activity of antibiotics treated in (**A**) water and (**B**) meat effluent after 0, 15 and 25 min of treatment tested against *E*. *coli* and *B*. *atrophaeus*. Results represent mean values of measured inhibition zone diameter, mm (n = 6). Different letters indicate a significant difference between the diameters (p < 0.05) of each bacterial group and type of antibiotic studied. Vertical bars represent standard deviation.

The degradation of antibiotics should be accompanied with the loss of biological function. The residual antibacterial activity of CFX and OFX dissolved in either water or meat effluent was examined using a disk diffusion assay and measuring the inhibition zone diameter (mm) for *E*. *coli*, *B*. *atrophaeus and P*. *aeruginosa* (Figs S6 and S7). It should be noted that all controls resulted in no inhibition zone formed around the disks and that the disk assay involving *P*. *aeruginosa* was not valid due to the resistance of this microorganism to studied antibiotics at the concentration obtained on disks (~0.2 μg disk^−1^). In both cases the inhibition zone diameter was recorded as 6 mm, which was equal to the diameter of the disk. In general, the efficacy of ACP treatment for the degradation of antibiotics was influenced by the duration of the treatment and was affected by the type of antibiotic and the sample matrix. The antibacterial activity of OFX and CFX is linked to the presence of carboxylic and carbonyl groups in the quinolone molecule. Thus, the antimicrobial activity of both antibiotics dissolved in water was significantly reduced (p < 0.05) after either 15 or 25 min of treatment as was recorded for the microorganisms tested (Fig. 7A). A lower degradation ability of ACP was observed when the antibiotics were dissolved in the meat effluent (Fig. 7B). Moreover, in this case the effect of the type of antibiotic on ACP degradation ability was more apparent, with CFX exhibiting higher resistance to ACP than OFX. The CFX mechanism of degradation also supports the results from the observed antibacterial activity. It was found that the quinolone moiety of OFX was completely transformed. However, the CFX transformation was reserved only for piperazine, fluorine substituents, with the core quinolone structure remaining intact. Therefore, the efficacy of plasma treatment for antibiotic degradation depends on the susceptibility of the core structure of the chemical compound to oxidation pathways. Plasma treatment may not sufficiently oxidize or mineralize the quinolone ring of CFX, but can cause significant transformations to the auxiliary functional groups, which could also decrease the antibacterial activity. A relationship between degradation efficiency and antimicrobial activity reduction was observed.

To confirm the degradation efficacy of plasma, the effect of the longest treatment time (25 min) on the antimicrobial activity of the antibiotics suspended in a simple sample matrix (water) was quantitatively assessed using the broth microdilution method. This estimated the MIC values for the three microorganisms selected (Supplementary Table S7). However, a relatively low agreement between the two tests was found in the case of the remaining antimicrobial activity of CFX. According to the MIC values for *E*. *coli*, *B*. *atrophaeus* and *P*. *aeruginosa*, the CFX samples exhibited a higher antimicrobial activity after ACP treatment than the corresponding untreated control. In contrast, higher MICs were recorded for ACP treated OFX as compared with MICs of the untreated control for all microorganisms tested. This indicates that the reduction of the antimicrobial activity of this antibiotic (OFX) is due to the plasma treatment, which is in agreement with the disk diffusion assay. Therefore, for OFX, the degradation processes did not generate by-products with a greater antimicrobial activity than the parent compound. These observations were also demonstrated by De Witte *et al*.^69^ for ozonation and by Paul *et al*.^70^ for UVA photo catalysis.

The increased activity of CFX highlights the generation of compounds more toxic to cells due to the plasma exposure, which was not reflected with the disk diffusion assay. Such discrepancy between the two techniques could be due to the decreased stability of the generated by-products that exhibit antimicrobial properties in the disk diffusion assay or impaired diffusion capacity of these compounds into the agar. The antimicrobial potential of the water (antibiotic diluent) subjected to plasma treatment was tested using a two-fold microdilution method to examine its potential microbial toxicity. Plasma treated water was not toxic to cells at concentration below 12.5% for *P*. *aeruginosa* and 6.2% for *E*. *coli* and *B*. *atrophaeus* that do not correspond to the dilutions at which MICs for the two antibiotics were reached (Supplementary Table S7). These results demonstrated that the retention of antimicrobial potential of treated antibiotics could not be attributed to the antimicrobial properties of the plasma treated water.

**Figure 8:**
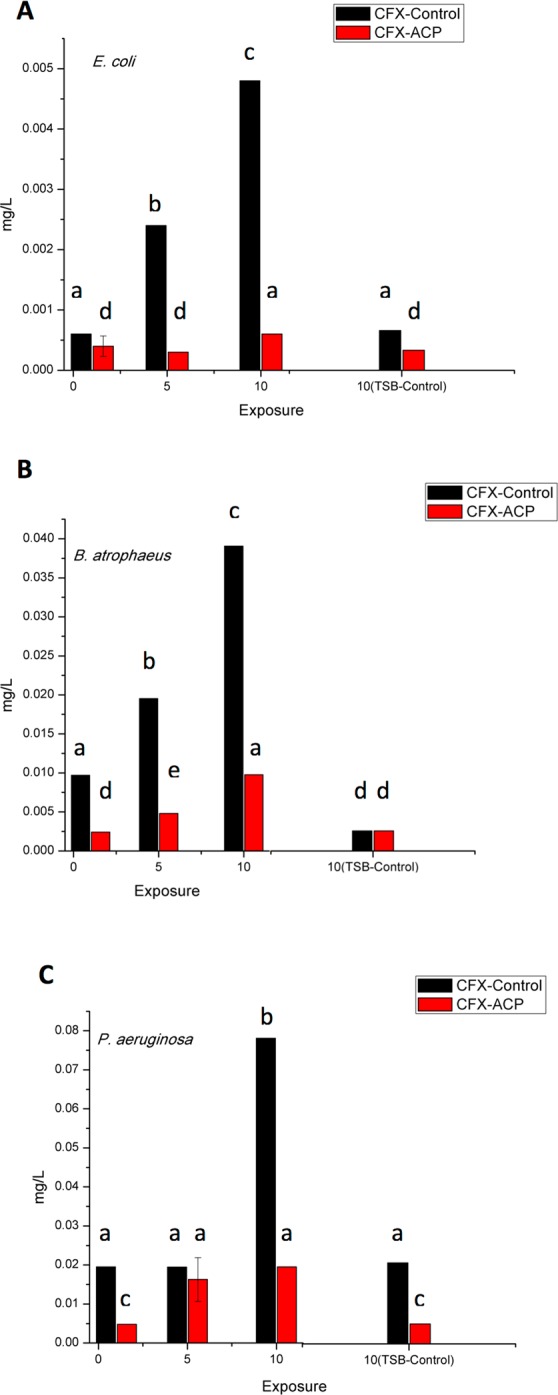
Minimum inhibitory concentration of either ACP treated or untreated control ciprofloxacin for *E*. *coli* (**A**), *B*. *atrophaeus* (**B**) and *P*. *aeruginosa* (**C**) exposed to corresponding antibiotic sample or TSB (TSB-control) for up to ten consecutive exposures. Results represent mean values of estimated MIC, mg l^−1^ (n = 9). Different letters indicate a significant difference between MIC values (p < 0.05) obtained for each bacterial group. Vertical bars represent standard deviation.

Unintentional exposure of microorganisms to sub-inhibitory concentrations of biocides in the environment can lead to the development of antimicrobial resistance, which is a global healthcare problem^71^. Because of the demonstrated increase in the antimicrobial potency of ACP treated CFX, a study on bacterial adaptation to CFX was conducted. For *E*. *coli*, the tendency of bacterial adaptation after repeated exposure to untreated CFX was observed, with a significant, 8-fold increase in MIC recorded (p < 0.05). Importantly, there was only 1.5-fold increase in MIC value noted in the case of ACP treated antibiotic (Fig. 8A), which is a useful observation indicating synergistic effects of antibiotics and plasma treatment. Such an effect could improve the efficiency of conventional antimicrobials and/or antibiotic treatments. A significant reduction (p < 0.05) in susceptibility to both ACP treated and untreated CFX was observed for *B*. *atrophaeus* and *P*. *aeruginosa*, with a 4-fold increase in the MIC values found (Fig. 8B,C, respectively). This study revealed that short-term exposure to sub-lethal concentrations of antibiotics present in effluents, whether they were treated with cold plasma or not, equally reduced antibiotic susceptibility, which could potentially select for bacterial populations with stable genetic mutations. Therefore, it is recommended that cold plasma as an AOP for industrial effluents that may contain antibiotics or residues is optimized for complete loss of biological activity or is combined with a separate process to ensure life cycle safety.

In conclusion, we have determined the optimum operating conditions for the degradation of antibiotics (OFX and CFX) using an atmospheric air plasma DBD plasma reactor. The results revealed that the rates of the degradation order were dependent on treatment time, voltage as well as sample matrix. The degradation rate was more pronounced for OFX than CFX due to differences in their chemical structure of antibiotics. The reduced TOC removal values indicates that the antibiotics and organic matter are degraded to smaller molecules such as NO_3_, acetic and formic acids. The degradation pathway includes the oxidation of piperazinyl and quinolone moieties. With the decrease of antibiotic concentration, antibacterial activity also decreased according to results obtained by using the disk diffusion assay, showing that there is a relationship between degradation and antibacterial activity reduction. There was no major difference observed between the adaptations of the microorganisms to treated or untreated antibiotics. Plasma treatment could be a promising addition to existing advanced oxidation processes, as efficient degradation of antibiotics in industrial effluents can be achieved. However, further studies are required in order to investigate the potential for genetic mutation responses as well as eco-toxicological effects of plasma treated antibiotic solutions in the form of individual compounds as well as complex mixtures, when such solutions and their degradants are released into the environment.

## Methods

### Materials

Analytical grade standards of ofloxacin (OFX) and ciprofloxacin (CFX) of purity (>98%), HPLC grade methanol, acetonitrile, ethyl acetate, ammonium hydroxide solution (32%) puriss p.a. (NH_4_OH), acetic acid (AcOH), formic acid (HCOOH), sodium acetate (NaOAc), ammonium formate, tertiary butanol alcohol (TBA), carbon tetrachloride (CCl_4_) and LC-MS grade water were obtained from Sigma-Aldrich (Ireland).

### Sample Preparation

A fresh model effluent was prepared before each experimental run using procedure of Barrera *et al*.^72^. The raw synthetic meat effluent consisted of distilled water, commercial meat extract powder 1950 mg l^−1^; glycerol (C_3_H_8_O_3_), 200 mg l^−1^; ammonium chloride (NH_4_Cl), 360 mg/l and sodium chloride (NaCl) 50 mg l^−1^. The model effluent was used to overcome the inherent variability found in commercial effluent composition. Large particulate matter was removed by filtering the model effluent through a Whatman (UK) filter paper and a 0.45 μm membrane (Millipore). Because of the structural characteristics and persistence in the environment, ofloxacin (OFX) and ciprofloxacin (CFX) were selected in this work. Each antibiotic (OFX and CFX) was dissolved in methanol to obtain a standard stock solution with the concentration 1000 mg l^−1^. The prepared stock solution was either diluted in water or spiked with the model effluent to obtain a minimum concentration of antibiotic 10 mg l^−1^.

### Atmospheric air cold plasma treatment

The high voltage in package ACP DBD system employed for this work is described in Sarangapani *et al*.^73^ and was fully characterized in Moiseev *et al*.^74^ and Patil *et al*.^75^. The schematics of the system is presented in Supplementary Fig. S8. For each experiment 25 ml of water or meat effluent spiked with antibiotics at initial concentration of 10 mg l^−1^ was subjected to ACP treatment. Atmospheric air was used as the working gas. Plasma treatment was performed at variable voltage (70–80 kV) and treatment duration (5–25 min). Treatment was carried out in duplicate at ambient temperature (~18 °C). The temperature increase inside the container and at the surface of the samples due to plasma treatment was <5 °C. After processing, containers were stored at room temperature of ~18 °C for 24 h in line with our previous findings that post treatment retention time is useful for biocontrol. This treatment approach allows extended contact time of the generated and contained chemical reactive species with the samples. Control samples were not plasma treated. Ozone concentrations were measured using short-term ozone detection tubes obtained from Gastec (Product No. 18 M, Gastec, Japan), while dissolved ozone measurements were carried out according to the procedure of Bader^76^.

### Analytical Methods

Standard curves for the antibiotics were established using standard solutions ranging between 0.01 mg l^−1^ and 10 mg l^−1^. The linear correlation coefficients (r^2^) were 0.997 and 0.998 for OFX and CFX, respectively. The plasma treated effluents were firstly extracted by solid-phase extraction (SPE) according procedure of Sarangapani *et al*.^49^. Antibiotics quantification was performed using a HPLC system (Waters, Ireland). The mobile phase consisted of 70% acetonitrile and of 30% 0.03 M phosphate buffer solution at pH 2.7, and the flow rate was set at 1 ml min^−1^. The detector wavelength was set at 277 nm. Chromatographic data was collected and processed using Empower2 software (Waters, Ireland).

The intermediates from plasma discharge were analyzed by LC-MS/MS coupled to a triple quadrupole instrument operating in electrospray ionization (ESI) mode (Agilent, Ireland) Milford, MA, USA). Separation was carried out on Phenomenex Gemini-Nx C18 column (Phenomenex, U.K.), 5 μm particle size (250 mm × 4.6 mm) maintained at 30 °C. A binary gradient system was used to separate analytes. Mobile phase A consisted of 50 mM formic acid and 2 mM ammonium formate in water, while mobile phase B consisted of 50 mM formic acid and 2 mM ammonium formate in acetonitrile. The gradient profile was linear from 30% B to 50% B over 3 min and 100% B at 5 min, then 2 min at 30% B followed by 3 min for re-equilibration at 30% B. The LC-MS/MS system and data was processed by agilent mass hunter software. A full scan mode was used to acquire MS/MS spectra of the intermediates with a scan range of m/z 80–500. The organic acids in the effluent were quantified by an ion chromatograph (ICS 3000, Dionex, USA) at 30 °C. Hydrogen peroxide and nitrate concentrations were determined according to procedure of Boehm *et al*.^48^ and Lu *et al*.^53^ Briefly, hydrogen peroxide concentration was determined by oxidation of potassium iodide (KI) to iodine and measured spectrophotometrically at 405 nm using 96 well plate. Either plasma treated samples, untreated controls or standard curve samples (50 μl), phosphate buffer (50 μl) and KI (100 μl) were added to each well. After 20 min incubation at room temperature absorbance was measured. A standard curve with known hydrogen peroxide concentration was included on the same plate to convert absorbance into peroxide concentration. Nitrite concentration was measured by Griess reagent (Sigma-Aldrich) in 96 well plates. Griess reagent (50 μl) was added to either treated, untreated or standard samples (50 μl). After 30 min incubation, absorbance was measured at 548 nm. A range of nitrite solutions of known concentrations were prepared to make the nitrite calibration curve, which was used to convert absorbance into nitrite concentrations. Nitrate concentrations were determined photometrically by 2,6-dimethyl phenol (DMP) using the Spectroquant nitrate assay kit (Merck 1.09713, Darmstadt, Germany) and calculated using a NaNO_3_ standard curve. Tests were performed using 96 well plates. In addition, the contribution of radical scavengers to the degradation efficiency of antibiotics was investigated by adding tBuOOH (tertiary butyl alcohol is considered as an affective hydroxyl radical scavenger) and CCl_4_ (hydrogen radical scavenger) to the aqueous antibiotics solutions^77^.

### Degradation kinetic modeling study and data analysis

The removal efficiencies (*η*) of antibiotics were calculated according to the following equation: *η = (C*_0_ *−* *C)*/*C*_0_ × *100*, where *η* is removal efficiency of each antibiotic, $C$ is the concentration of antibiotic at time ‘t’ and ${C}_{0}$ the initial concentration of the antibiotic. Plasma degradation of antibiotics in aqueous media can be described by the following equation: *C = C*_*0*_ *exp (−kt)*, where ‘*k*’ is the degradation rate constant (min^−1^) of the reaction and *‘t’* is the treatment time (min).

### Antimicrobial activity

#### Disk diffusion assay

To determine the remaining antimicrobial activity of tested antibiotics (OFX and CFX), standard disk diffusion assay was utilized involving three single species test microorganisms, namely *Escherichia coli* ATCC 25922, *Bacillus atrophaeus* var. *niger* (Sportrol®/Namsa®, VWR International, ATCC 9372) and *Pseudomonas aeruginosa* ATCC 27853, obtained from School of Food Science and Environmental Health of the Dublin Institute of Technology. Each bacterium was inoculated in tryptic soy broth (TSB, Scharlau Chemie, Spain) and incubated overnight at 37 °C. The density of each overnight culture was adjusted to McFarland 0.5. Resulting cell suspensions were further used for inoculation of tryptic soy agar (TSA, Biokar Diagnostics, France). Either ACP treated (15 and 25 min) or non-treated antibiotic solutions were filtered (0.2 μm pore size) and 20 μl aliquots were deposited on sterile discs (diameter 6 mm, Whatman®), while either ACP treated or untreated sterile deionized water and meat effluent served as a control. The disks were dried inside the laminar flow biosafety cabinet and placed on inoculated TSA plates. Following incubation for 24 h at 37 °C the zone of inhibition (diameter, mm) produced around the disks was measured at the point where no growth was observed. Experiments were performed in triplicate and replicated twice (n = 6).

#### Minimum inhibitory concentration (MIC)

MIC of either treated (ACP treatment − 25 min) or untreated antibiotic solutions in water for *E*. *coli*, *B*. *atrophaeus* and *P*. *aeruginosa* were determined using broth microdilution method^78^. Controls included each antibiotics concentration without bacterial cells (blank), TSB and bacterial cells (positive control) and two-fold diluted plasma treated water in TSB with final concentration of plasma treated water ranging from 50–0.01%. Plates were incubated for 24 h at 37 °C. MICs of either treated or untreated antibiotic for three microorganisms were determined as the lowest concentration showing no turbidity as comparing to corresponding blank controls. Plasma treatment experiments were replicated three times and two biological replicates were included in each plate (n = 6). The lowest antibiotic concentration that inhibited the growth of the microorganism was detected by the lack of visual turbidity (matching corresponding blank control) and was designated as MIC^79^.

#### Bacterial adaptation study

To study the possible development of resistance to ACP treated CFX, three biological replicates of each microorganism (*E*. *coli*, *B*. *atrophaeus* and *P*. *aeruginosa*) and three independently treated antibiotic in water (ACP treatment − 25 min) were used (n = 9). Overnight cultures were adjusted to ~6 log_10_ colony forming units (CFU) ml^−1^ in TSB. Either treated or untreated antibiotics were filter sterilized (pore size 0.2 μm) and twofold dilution series were prepared in 96 well plates with highest concentration corresponding to the double of the lowest MIC value established and the lowest concentration corresponding to 1/8 MIC. Bacterial cell suspensions (50 μl) were added to resulting concentrations of each antibiotic (50 μl). TSB without antibiotics was used as a control. Plates were incubated for 24 h at 37 °C. After incubation, MICs for each antibiotic were recorded and bacterial samples from the well containing sub-inhibitory concentration corresponding to ¼MIC were streaked on TSA^80^. The TSA plates were incubated for 24 h at 37 °C. Using isolated colonies the concentration of cells was visually adjusted with saline (0.85% NaCl) to a turbidity equivalent to the McFarland 0.5 standard. Bacterial suspensions were further diluted in TSB to a final cell concentration of ~6 log_10_ CFU ml^−1^. Again, aliquots of bacterial inoculum (50 μl) were dispensed into the wells containing doubling dilutions of each antibiotic with final absorbance measured at 600 nm corresponding to ~0.05. Viable count of the inoculum suspension using TSB without antibiotics was performed each time to ensure that final concentration of the inocula in the wells corresponds to ~5 log_10_ CFU ml^−1^. This procedure was repeated for ten consecutive cycles. Bacterial cells recovered after 1^st^, 5^th^ and 10^th^ exposures to sub-lethal doses of antibiotics were used to determine the MIC values, which were compared with MICs determined for cells exposed to TSB.

### Statistical analysis

Statistical analysis was performed using IBM SPSS statistics 21 Software (SPSS Inc., Chicago, USA). The values of the inhibition zone diameter measured for either untreated control or ACP treated for 15 and 25 min antibiotic samples for *E*. *coli*, *B*. *atrophaeus* and *P*. *aeruginosa* and MIC of CFX for each bacteria in bacterial adaptation study were subjected to Analysis Of Variance (ANOVA). Means of the diameters and MICs were compared according to the method of Fisher’s Least Significant Difference-LSD at the 0.05 level for each type of bacteria and antibiotic group.

## Supplementary information

Supplemetary materials

## Data Availability

All data generated or analysed during this study are included in this published article (and its Supplementary Information files).

## References

[1] Brain, R. A., Hanson, M. L., Solomon, K. R. & Brooks, B. W. Reviews of Environmental Contamination and Toxicology. **192** (2008). 10.1007/978-0-387-71724-1_3 18020304

[2] Fatta-Kassinos D, Meric S, Nikolaou A (2011). Pharmaceutical residues in environmental waters and wastewater: Current state of knowledge and future research. Anal. Bioanal. Chem..

[3] Tay KS, Madehi N (2015). Ozonation of ofloxacin in water: By-products, degradation pathway and ecotoxicity assessment. Sci. Total Environ..

[4] Sukul P, Spiteller M (2007). Fluoroquinolone antibiotics in the environment. Rev. Environ. Contam. Toxicol..

[5] Guerra P, Kim M, Shah A, Alaee M, Smyth SA (2014). Occurrence and fate of antibiotic, analgesic/anti-inflammatory, and antifungal compounds in five wastewater treatment processes. Sci. Total Environ..

[6] Michael I (2013). Urban wastewater treatment plants as hotspots for the release of antibiotics in the environment: A review. Water Res..

[7] Golet EM, Xifra I, Siegrist H, Alder AC, Giger W (2003). Environmental Exposure Assessment of Fluoroquiniolone Antibacterial Agents from Sewage to Soil. Environ. Sci. Technol..

[8] Zhang H, Liu P, Feng Y, Yang F (2013). Fate of antibiotics during wastewater treatment and antibiotic distribution in the effluent-receiving waters of the Yellow Sea, northern China. Mar. Pollut. Bull..

[9] Batt AL, Kim S, Aga DS (2007). Comparison of the occurrence of antibiotics in four full-scale wastewater treatment plants with varying designs and operations. Chemosphere.

[10] Carmosini N, Lee LS (2009). Ciprofloxacin sorption by dissolved organic carbon from reference and bio-waste materials. Chemosphere.

[11] Nasuhoglu D, Rodayan A, Berk D, Yargeau V (2012). Removal of the antibiotic levofloxacin (LEVO) in water by ozonation and TiO 2 photocatalysis. Chem. Eng. J..

[12] Carbajo JB (2015). Continuous ozonation treatment of ofloxacin: Transformation products, water matrix effect and aquatic toxicity. J. Hazard. Mater..

[13] Pérez-Moya M (2010). Characterization of the degradation performance of the sulfamethazine antibiotic by photo-Fenton process. Water Res..

[14] Gonçalves AG, órfão JJM, Pereira MFR (2012). Catalytic ozonation of sulphamethoxazole in the presence of carbon materials: Catalytic performance and reaction pathways. J. Hazard. Mater..

[15] Bhakta JN, Munekage Y (2009). Degradation of Antibiotics (Trimethoprim and Sulphamethoxazole) Pollutants Using UV and TiO2 in Aqueous Medium. Mod. Appl. Sci..

[16] Yuan F (2011). Photodegradation and toxicity changes of antibiotics in UV and UV/H2O2process. J. Hazard. Mater..

[17] Ben W, Qiang Z, Pan X, Nie Y (2012). Degradation of Veterinary Antibiotics by Ozone in Swine Wastewater Pretreated with Sequencing BatchReactor. J. Environ. Eng..

[18] Lange F (2006). Degradation of macrolide antibiotics by ozone: A mechanistic case study with clarithromycin. Chemosphere.

[19] Klavarioti M, Mantzavinos D, Kassinos D (2009). Removal of residual pharmaceuticals from aqueous systems by advanced oxidation processes. Environ. Int..

[20] Gómez-Ramos M (2011). Chemical and toxicological evolution of the antibiotic sulfamethoxazole under ozone treatment in water solution. J. Hazard. Mater..

[21] Dantas RF (2007). Bezafibrate removal by means of ozonation: Primary intermediates, kinetics, and toxicity assessment. Water Res..

[22] Rosal R (2009). Identification of intermediates and assessment of ecotoxicity in the oxidation products generated during the ozonation of clofibric acid. J. Hazard. Mater..

[23] Stalter D, Magdeburg A, Oehlmann J (2010). Comparative toxicity assessment of ozone and activated carbon treated sewage effluents using an *in vivo* test battery. Water Res..

[24] Jiang B (2014). Review on electrical discharge plasma technology for wastewater remediation. Chem. Eng. J..

[25] Jiang, B., Zheng, J. & Wu, M. *Nonthermal Plasma for Effluent and Waste Treatment*. *Cold Plasma in Food and Agriculture: Fundamentals and Applications*, 10.1016/B978-0-12-801365-6.00013-5 (2016).

[26] Bourke, P., Ziuzina, D., Boehm, D., Cullen, P. J. & Keener, K. The Potential of Cold Plasma for Safe and Sustainable Food Production. *Trends Biotechnol*, 10.1016/j.tibtech.2017.11.001 (2018). 10.1016/j.tibtech.2017.11.001 29329724

[27] Sarangapani C (2017). Optimization of atmospheric air plasma for degradation of organic dyes in wastewater. Water Sci. Technol..

[28] Sarangapani C (2016). Pesticide degradation in water using atmospheric air cold plasma. J. Water Process Eng..

[29] Sarangapani C, O’Toole G, Cullen PJ, Bourke P (2017). Atmospheric cold plasma dissipation efficiency of agrochemicals on blueberries. Innov. Food Sci. Emerg. Technol..

[30] Devi Y, Thirumdas R, Sarangapani C, Deshmukh RR, Annapure US (2017). Influence of cold plasma on fungal growth and aflatoxins production on groundnuts. Food Control.

[31] Ziuzina, D., Patil, S., Cullen, P. J., Keener, K. M. & Bourke, P. Atmospheric cold plasma inactivation of Escherichia coli in liquid media inside a sealed package. *J*. *Appl*. *Microbiol*. **114** (2013). 10.1111/jam.12087 23190122

[32] Patange A (2018). Assessment of the disinfection capacity and eco-toxicological impact of atmospheric cold plasma for treatment of food industry effluents. Sci. Total Environ..

[33] Shen, J. *et al*. Bactericidal Effects against S. aureus and Physicochemical Properties of Plasma Activated Water stored at different temperatures. *Sci*. *Rep*. **6** (2016). 10.1038/srep28505 PMC4921907 27346695

[34] Shaw P (2018). Bacterial inactivation by plasma treated water enhanced by reactive nitrogen species. Sci. Rep..

[35] Kim KS, Kam SK, Mok YS (2015). Elucidation of the degradation pathways of sulfonamide antibiotics in a dielectric barrier discharge plasma system. Chem. Eng. J..

[36] Magureanu M (2011). Degradation of antibiotics in water by non-thermal plasma treatment. Water Res..

[37] Lou J, Lu N, Li J, Wang T, Wu Y (2012). Remediation of chloramphenicol-contaminated soil by atmospheric pressure dielectric barrier discharge. Chem. Eng. J..

[38] Kim KS, Yang CS, Mok YS (2013). Degradation of veterinary antibiotics by dielectric barrier discharge plasma. Chem. Eng. J..

[39] Magureanu M (2010). Degradation of pharmaceutical compound pentoxifylline in water by non-thermal plasma treatment. Water Res..

[40] Magureanu M, Mandache NB, Parvulescu VI (2015). Degradation of pharmaceutical compounds in water by non-thermal plasma treatment. Water Res..

[41] Rong SP, Sun YB, Zhao ZH (2014). Degradation of sulfadiazine antibiotics by water falling film dielectric barrier discharge. Chinese Chem. Lett..

[42] Liu Y, Mei S, Iya-Sou D, Cavadias S, Ognier S (2012). Carbamazepine removal from water by dielectric barrier discharge: Comparison of *ex situ* and *in situ* discharge on water. Chem. Eng. Process. Process Intensif..

[43] Hama Aziz KH (2017). Degradation of pharmaceutical diclofenac and ibuprofen in aqueous solution, a direct comparison of ozonation, photocatalysis, and non-thermal plasma. Chem. Eng. J..

[44] Tripathi S, Tripathi BD (2011). Efficiency of combined process of ozone and bio-filtration in the treatment of secondary effluent. Bioresour. Technol..

[45] Lukes, P., Brisset, J. & Locke, B. R. Biological Effects of Electrical Discharge Plasma in Water and in Gas – Liquid Environments. 36–43 (2012).

[46] Wang J, Sun Y, Jiang H, Feng J (2017). Removal of caffeine from water by combining dielectric barrier discharge (DBD) plasma with goethite. J. Saudi Chem. Soc..

[47] Locke, B. R. & Shih, K. Y. Review of the methods to form hydrogen peroxide in electrical discharge plasma with liquid water. *Plasma Sources Sci*. *Technol*. **20** (2011).

[48] Boehm D, Heslin C, Cullen PJ, Bourke P (2016). Cytotoxic and mutagenic potential of solutions exposed to cold atmospheric plasma. Sci. Rep..

[49] Sarangapani C (2017). Efficacy and mechanistic insights into endocrine disruptor degradation using atmospheric air plasma. Chem. Eng. J..

[50] Reddy MKP, Mahammadunnisa S, Subrahmanyam C (2014). Catalytic non-thermal plasma reactor for mineralization of endosulfan in aqueous medium: A green approach for the treatment of pesticide contaminated water. Chem. Eng. J..

[51] Misra NN (2014). In-package nonthermal plasma degradation of pesticides on fresh produce. J. Hazard. Mater..

[52] Staehelin J, Holgné J (1982). Decomposition of Ozone in Water: Rate of Initiation by Hydroxide Ions and Hydrogen Peroxide. Environ. Sci. Technol..

[53] Lu, P., Boehm, D., Bourke, P. & Cullen, P. J. Achieving reactive species specificity within plasma-activated water through selective generation using air spark and glow discharges. *Plasma Process Polym*., 10.1002/ppap.201600207 (2016).

[54] Brisset JL, Hnatiuc E (2012). Peroxynitrite: A re-examination of the chemical properties of non-thermal discharges burning in air over aqueous solutions. Plasma Chem. Plasma Process..

[55] Brisset, J. L., Benstaali, B., Moussa, D., Fanmoe, J. & Njoyim-Tamungang, E. Acidity control of plasma-chemical oxidation: Applications to dye removal, urban waste abatement and microbial inactivation. *Plasma Sources Sci*. *Technol*. **20** (2011).

[56] Lukes, P., Dolezalova, E., Sisrova, I. & Clupek, M. Aqueous-phase chemistry and bactericidal effects from an air discharge plasma in contact with water: Evidence for the formation of peroxynitrite through a pseudo-second-order post-discharge reaction of H2O2 and HNO2. *Plasma Sources Sci*. *Technol*. **23** (2014).

[57] Gao L, Sun L, Wan S, Yu Z, Li M (2013). Degradation kinetics and mechanism of emerging contaminants in water by dielectric barrier discharge non-thermal plasma: The case of 17β-Estradiol. Chem. Eng. J..

[58] Panorel I, Preis S, Kornev I, Hatakka H, Louhi-Kultanen M (2013). Oxidation of aqueous pharmaceuticals by pulsed corona discharge. Environ. Technol..

[59] Vasquez MI, Hapeshi E, Fatta-Kassinos D, Kümmerer K (2013). Biodegradation potential of ofloxacin and its resulting transformation products during photolytic and photocatalytic treatment. Environ. Sci. Pollut. Res..

[60] Stratton GR (2017). Plasma-Based Water Treatment: Efficient Transformation of Perfluoroalkyl Substances in Prepared Solutions and Contaminated Groundwater. Environ. Sci. Technol..

[61] Clennan EL, Chen M-F, Greer A, Jensen F (1998). Experimental and Computational Evidence for the Formation of Iminopersulfinic Acids. J. Org. Chem..

[62] Greer A, Chen MF, Jensen F, Clennan EL (1997). Experimental and ab initio computational evidence for new peroxidic intermediates (iminopersulfinic acids). Substituent effects in the photooxidations of sulfenic acid derivatives. J. Am. Chem. Soc..

[63] Pi Y, Feng J, Song M, Sun J (2014). Degradation potential of ofloxacin and its resulting transformation products during Fenton oxidation process. Chinese Sci. Bull..

[64] Shen L (1989). Cooperative Drug-DNA Binding Model. Biochemistry.

[65] Salma A, Thoröe-Boveleth S, Schmidt TC, Tuerk J (2016). Dependence of transformation product formation on pH during photolytic and photocatalytic degradation of ciprofloxacin. J. Hazard. Mater..

[66] Ferdig M, Kaleta A, Buchberger W (2005). Improved liquid chromatographic determination of nine currently used (fluoro)quinolones with fluorescence and mass spectrometric detection for environmental samples. J. Sep. Sci..

[67] Sturini M (2012). Photolytic and photocatalytic degradation of fluoroquinolones in untreated river water under natural sunlight. Appl. Catal. B Environ..

[68] Torniainen K, Mattinen J, Askolin CP, Tammilehto S (1997). Structure elucidation of a photodegradation product of ciprofloxacin. Journal of Pharmaceutical and Biomedical Analysis.

[69] De Witte B (2010). Ciprofloxacin ozonation in hospital wastewater treatment plant effluent: Effect of pH and H2O2. Chemosphere.

[70] Paul T, Dodd MC, Strathmann TJ (2010). Photolytic and photocatalytic decomposition of aqueous ciprofloxacin: Transformation products and residual antibacterial activity. Water Res..

[71] Abhilash NT (2012). Pharmaceuticals in Environment: A review on its effect. Res. J. Chem. Sci..

[72] Barrera M (2012). Photolytic treatment of organic constituents and bacterial pathogens in secondary effluent of synthetic slaughterhouse wastewater. Chem. Eng. Res. Des..

[73] Sarangapani C, Ryan Keogh D, Dunne J, Bourke P, Cullen PJ (2017). Characterisation of cold plasma treated beef and dairy lipids using spectroscopic and chromatographic methods. Food Chem..

[74] Moiseev, T. *et al*. Post-discharge gas composition of a large-gap DBD in humid air by UV-Vis absorption spectroscopy. *Plasma Sources Sci*. *Technol*. **23** (2014).

[75] Patil S (2014). Influence of high voltage atmospheric cold plasma process parameters and role of relative humidity on inactivation of Bacillus atrophaeus spores inside a sealed package. J. Hosp. Infect..

[76] Bader H (1982). Determination of Ozone In Water By The Indigo Method: A Submitted Standard Method. Ozone Sci. Eng..

[77] Sarangapani C, Lu P, Behan P, Bourke P, Cullen P (2018). Humic acid and trihalomethane breakdown with potential by-product formations for atmospheric air plasma water treatment. J. Ind. Eng. Chem..

[78] Balouiri M, Sadiki M, Ibnsouda SK (2016). Methods for *in vitro* evaluating antimicrobial activity: A review. J. Pharm. Anal..

[79] Ramalivhana J (2014). Antibacterial activity of honey and medicinal plant extracts against Gram negative microorganisms. African J. Biotechnol..

[80] Brun, P. *et al*. Antibacterial Efficacy and Mechanisms of Action of Low Power Atmospheric Pressure Cold Plasma: Membrane Permeability, Biofilm Penetration, and Antimicrobial Sensitization. *J*. *Appl*. *Microbiol*. 0–2 (2018). 10.1111/jam.13780 29655267

