# Supplementary material for: Degradation kinetics of cold plasma-treated antibiotics and their antimicrobial activity
Source: Sci Rep. 2019 Mar 8;9:3955. doi: 10.1038/s41598-019-40352-9 (PMC6408491; doi:10.1038/s41598-019-40352-9)
Supplement: Supplementary file 1 — Supplemetary materials [file 41598_2019_40352_MOESM1_ESM.docx]

**Degradation kinetics of cold plasma-treated antibiotics and their antimicrobial activity**

Chaitanya Sarangapani^1+^, Dana Ziuzina^1+*^, Patrice Behan^2^, Daniela Boehm^1^, Brendan F. Gilmore^3^, PJ Cullen^1,4^, Paula Bourke^1^.

**Supplementary material**

**Supplementary Table S1**

**Table S1: Model parameters for antibiotics based on first-order kinetics**

| Antibiotic | Voltage (kV) | Rate constant *k* (min^-1^) | R^2^ (Adj) |
| --- | --- | --- | --- |
| Water | | | |
| Ciprofloxacin | 70 | 0.0396±0.0048 | 0.95 |
|  | 80 | 0.0546±0.0084 | 0.90 |
| Ofloxacin | 70 | 0.089±0.0142 | 0.91 |
|  | 80 | 0.0926±0.0097 | 0.94 |
| Meat effluent | | | |
| Ciprofloxacin | 70 | 0.0458±0.0025 | 0.97 |
|  | 80 | 0.0636±0.0048 | 0.97 |
| Ofloxacin | 70 | 0.0522±0.0037 | 0.96 |
|  | 80 | 0.0671±0.0041 | 0.97 |

**Supplementary Figure S2** Proposed degradation pathway of ofloxacin (pathway I).


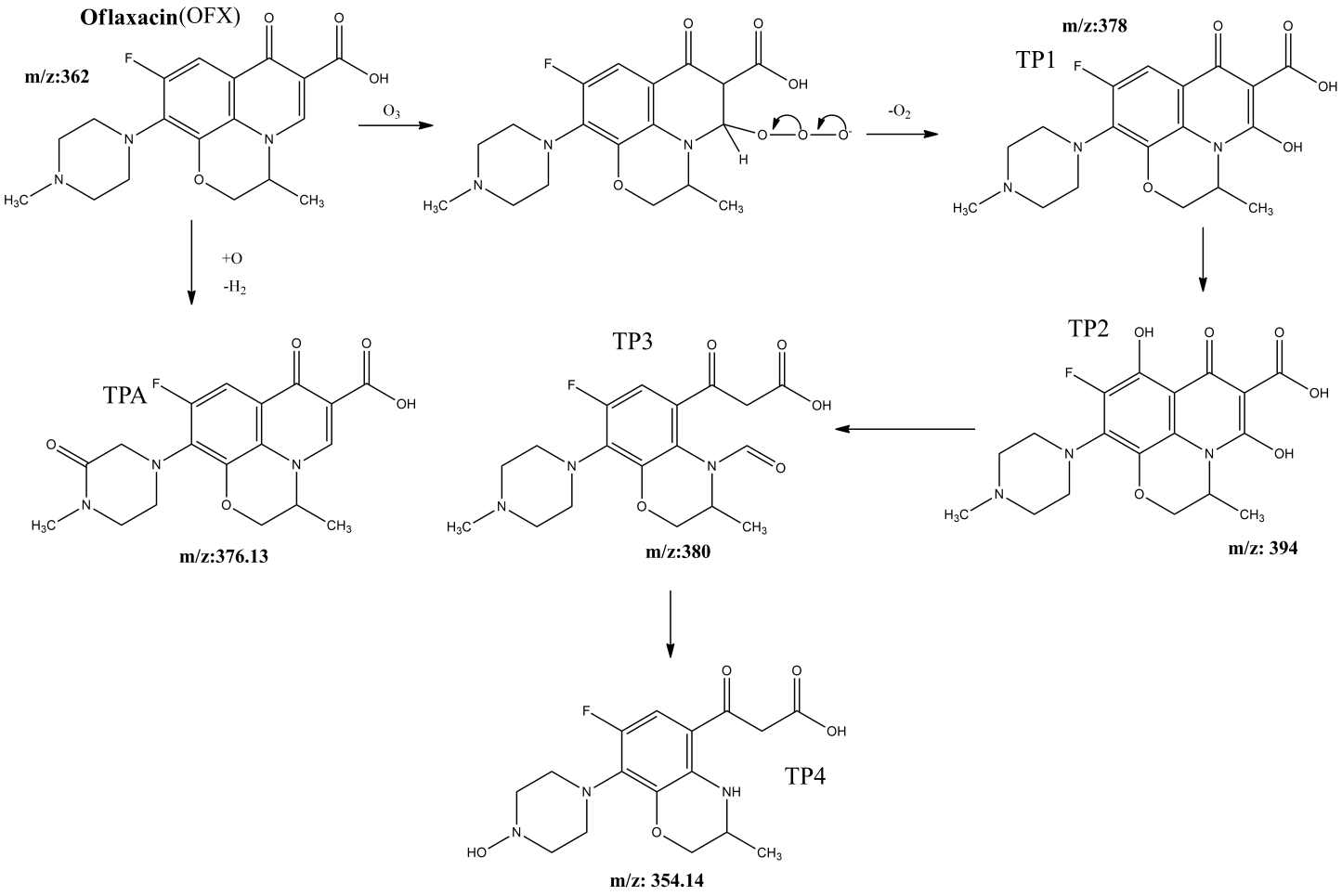
**Fig. S2.** Proposed degradation pathway of ofloxacin (pathway I).

**Supplementary Figure S3** Proposed degradation pathway of ofloxacin (pathway II)


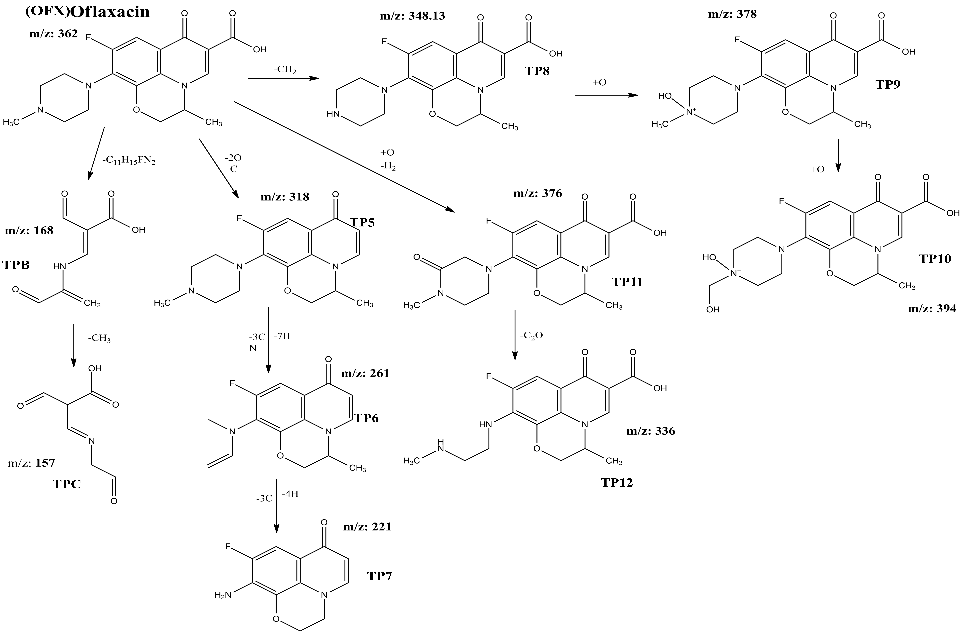


**Fig. S3.** Proposed degradation pathway of ofloxacin (pathway II).

**Supplementary Figure S4** Proposed degradation pathway of ciprofloxacin (pathway I).


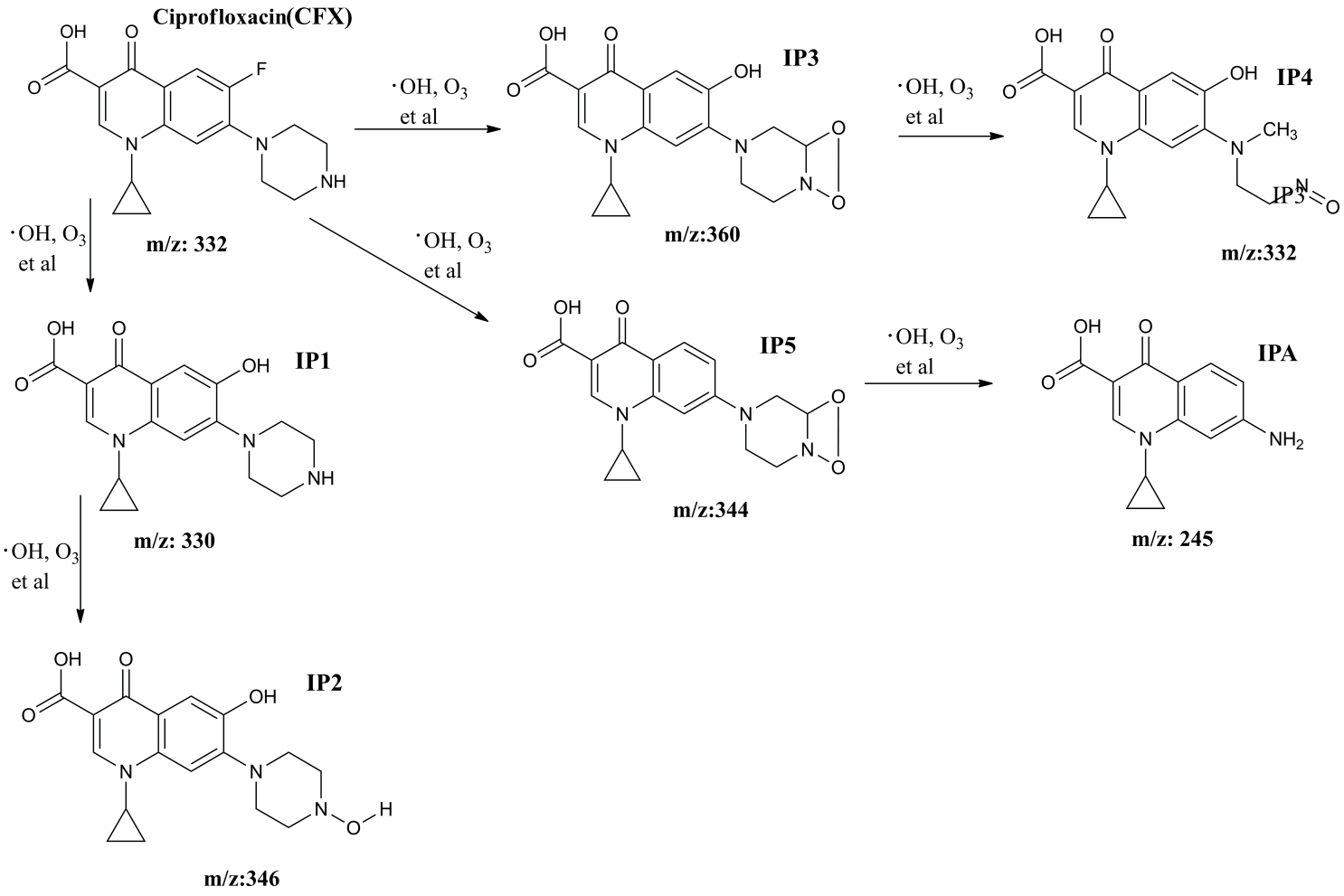
**Fig. S4.** Proposed degradation pathway of ciprofloxacin (pathway I).

**Supplementary Figure S5** Proposed degradation pathway of ciprofloxacin (pathway II).


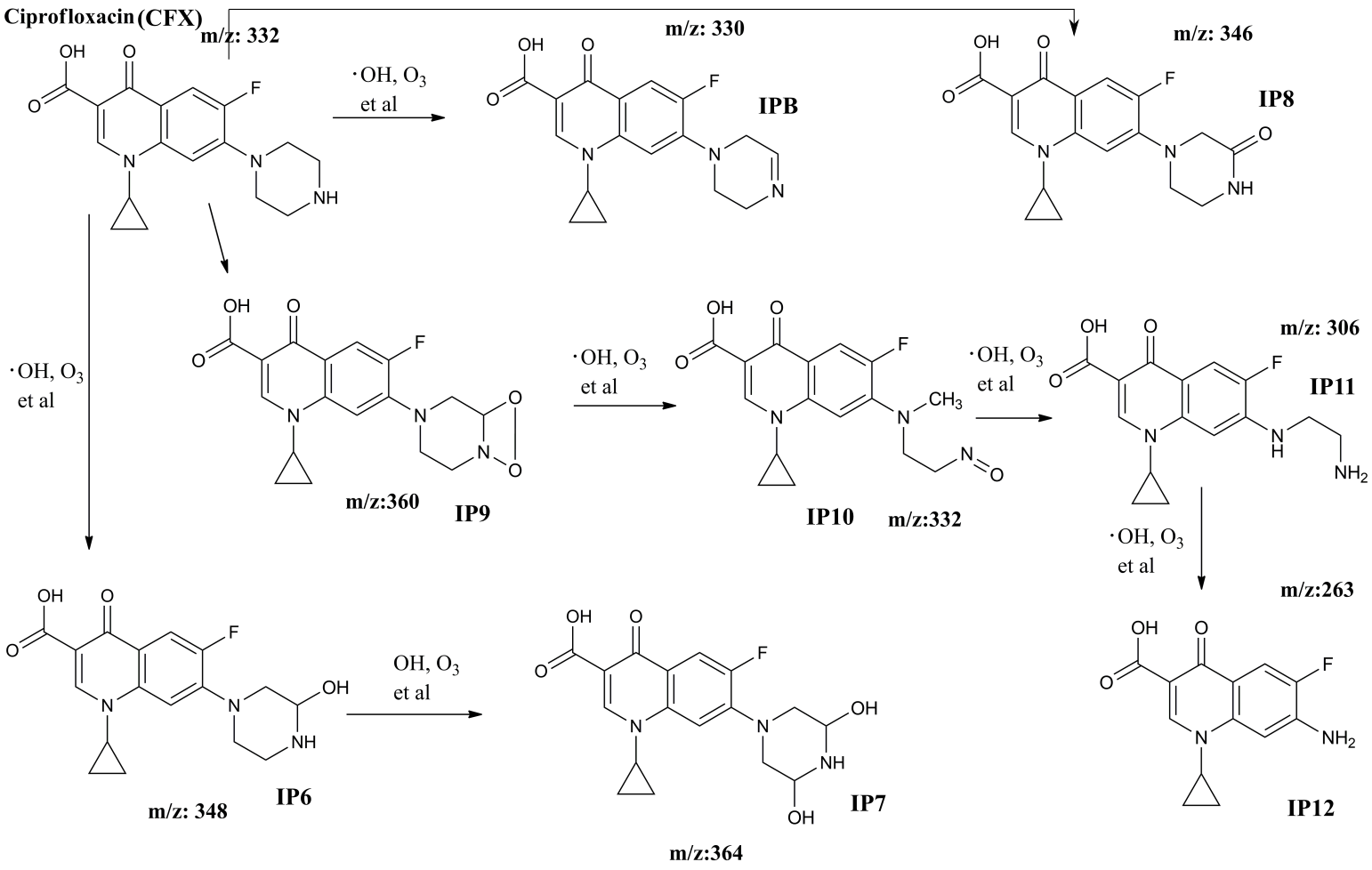


**Fig. S5.** Proposed degradation pathway of ciprofloxacin (pathway II).

**Supplementary Figure S6** A photograph containing four tryptic soy agar (TSA) plates, which represent disk diffusion assay performed for *B. atrophaeus* (top plates) and *E. coli* (bottom plates) to estimate inhibition zone (a clear zone formed around the disk) diameter for either treated or untreated ofloxacin (left top and bottom plate) and ciprofloxacin (right top and bottom plate) dissolved in water.


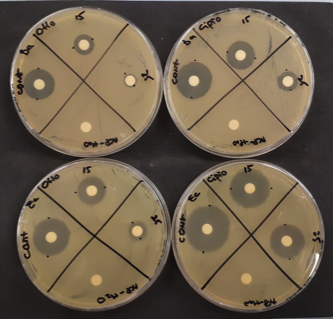


**Fig. S6.** A photograph of four TSA plates; each divided into four sections. Each section contains one disk with either control (plasma treated antibiotic solvent), ACP treated for 15/25 min antibiotic solution or untreated antibiotic. Top left plate – *B. atrophaeus* lawn formed in the presence of disks containing ofloxacin: left - untreated control, top - treated for 15 min, right - treated for 25 min and water treated for 15 min. Top right plate – *B. atrophaeus* lawn formed in the presence of disks containing ciprofloxacin: left - untreated control, top - treated for 15 min, right - treated for 25 min and water treated for 25 min. Bottom left plate – *E. coli* lawn formed in the presence of disks containing ofloxacin: left - untreated control, top - treated for 15 min, right - treated for 25 min and water treated for 15 min. Bottom right plate – *E. coli* lawn formed in the presence of disks containing ciprofloxacin: left - untreated control, top - treated for 15 min, right - treated for 25 min and water treated for 25 min.

**Supplementary Table S7**

**Table S6: Minimum inhibitory concentrations of untreated control and ACP treated (25 min) ciprofloxacin and ofloxacin in water**

| **Minimum inhibitory concentration (MIC) mg l^-1^** | | | | | | |
| --- | --- | --- | --- | --- | --- | --- |
|  | ***P. aeruginosa*** | | ***E. coli*** | | ***B. atrophaeus*** | |
|  | **Control** | **ACP** | **Control** | **ACP** | **Control** | **ACP** |
| **Ciprofloxacin** | 0.0195 | 0.0049-0.0195 | 0.0006-0.0049 | 0.0003-0.0012 | 0.0098-0.0195 | 0.0024-0.0098 |
| **Ofloxacin** | 0.0390 | 0.0390-0.1562 | 0.0006-0.0012 | 0.0012-0.0049 | 0.0024-0.0049 | 0.0049-0.0098 |

**Supplementary Figure S8** Schematic diagram of in package high voltage dielectric barrier discharge (DBD) atmospheric cold plasma (ACP) system.

**Fig. S8.** Schematics of in package high voltage DBD ACP system.
